# Combining X‑ray Diffraction, Solid-State NMR, and Computational Chemistry for Structural Insights and Performance Evaluation of GIPAW/GIAO ^13^C Chemical-Shift Calculations for Lamivudine and Three Pharmaceutical Salts

**DOI:** 10.1021/acsomega.6c03134

**Published:** 2026-09-02

**Authors:** Jefferson R. Dias-Silva, Vinícius S. Ferreira, Felipe T. Martins, Valdemar Lacerda Júnior, Pedro L. de Souza, Thiago M. F. Marinho, Luiz H. K. Queiroz Júnior

**Affiliations:** † Instituto de Química, 67824 Universidade Federal de Goiás, CP 131, 74001-970 Goiânia-GO, Brazil; ‡ Instituto Federal de Goiás, R. 75 n. 46, 74055-110 Goiânia-GO, Brazil; § Instituto de Química, Universidade Federal de Alfenas, 37130-001 Alfenas, MG, Brazil; ∥ Departamento de Química, Centro de Ciências Exatas, 28126 Universidade Federal do Espírito Santo, 29075-910 Vitória-ES, Brazil

## Abstract

The increasing prevalence of polymorphism and multicomponent crystal forms in active pharmaceutical ingredients demands rigorous structural characterization tools for quality control. Here, lamivudine and three pharmaceutical salts, hydrochloride, salicylate monohydrate, and hydrogen phthalate, were investigated by combining powder X-ray diffraction, ^13^C solid-state NMR spectroscopy, and two DFT-based methods for theoretical chemical-shift prediction: gauge including projected augmented wave (GIPAW) and gauge including atomic orbital (GIAO). In statistical comparison of experimental and theoretical chemical shifts, the GIPAW method consistently outperformed GIAO for all four crystalline forms, as evidenced by lower median errors and higher Kendall’s τ correlation coefficient values, a rank-order metric that proved more discriminating than Pearson’s *R* alone. The superior performance of GIPAW is attributed to its explicit treatment of periodic boundary conditions, which faithfully reproduces the crystallographic environment of the solid state. In addition, QTAIM topological analysis and natural bond orbital (NBO) second-order perturbation theory were used to characterize all inter- and intramolecular hydrogen bonds in each form. Two strong, nearly symmetric O···H···O interactions in the hydrogen phthalate salt are reported here for the first time, consistent with the anomalously low p*K*_a2_ of *ortho*-phthalic acid. These results demonstrate that the combined use of periodic GIPAW NMR calculations with QTAIM/NBO analysis constitutes a powerful, generally applicable strategy for the unequivocal structural characterization of pharmaceutical solid forms, with direct implications for the physicochemical quality control of drug substances.

## Graphical abstract

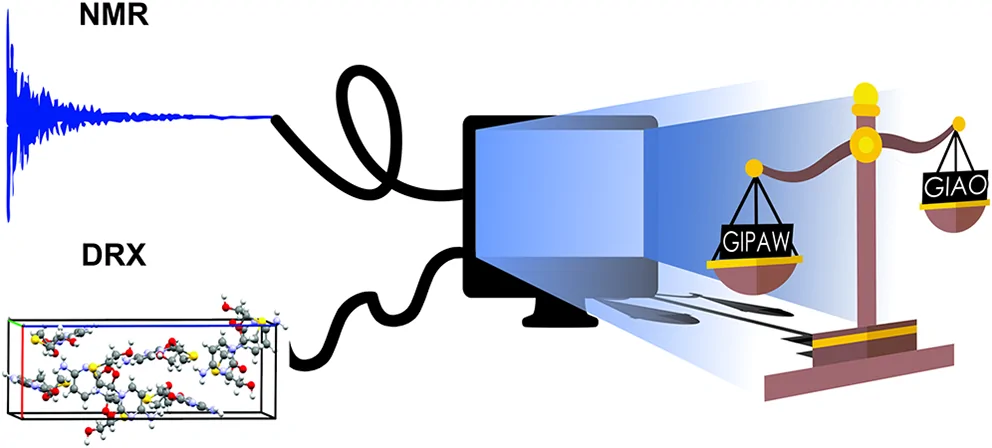

## 1 Introduction

The increasing incidence of solid-state polymorphism in drugs requires regulatory agencies to obtain extensive knowledge of new drugs to ensure the quality of existing crystalline forms and to minimize possible risks to public health.^1^ To this end, prior to the formulation of the active pharmaceutical ingredient (API) into a solid dosage form, it is important that the API is characterized chemically and physically, with the inclusion of analytical preformulation studies. This characterization can be performed by associating several solid-state techniques, such as powder X-ray diffraction (PXRD) crystallography, mid-infrared, and Nuclear Magnetic Resonance (NMR) spectroscopy. For solid dosage forms, the performance of the final medicine is entirely related to the crystal structure of the API, since different intermolecular arrangements result in different lattice energies. Consequently, an API can have different dissolution kinetics and equilibrium solubility according to its crystal form, which, in an ultimate analysis, will thus lead to changeable pharmacological performance.^2,3^

Lamivudine has been marketed worldwide by GlaxoSmithKline for more than two decades as one of the most widespread antiviral agents for the treatment of HIV/HBV. Lamivudine can present multiple crystalline forms; however, its anhydrated Form II is the only crystal form considered to be a true polymorph. It crystallizes^4,5^ in the tetragonal space group *P*4_3_2_1_2 with eight crystallographically equivalent drug molecules per cell unit (Figure 1), and it is this crystalline phase that is used in pharmaceutical preparations.^6–8^

**Figure 1:**
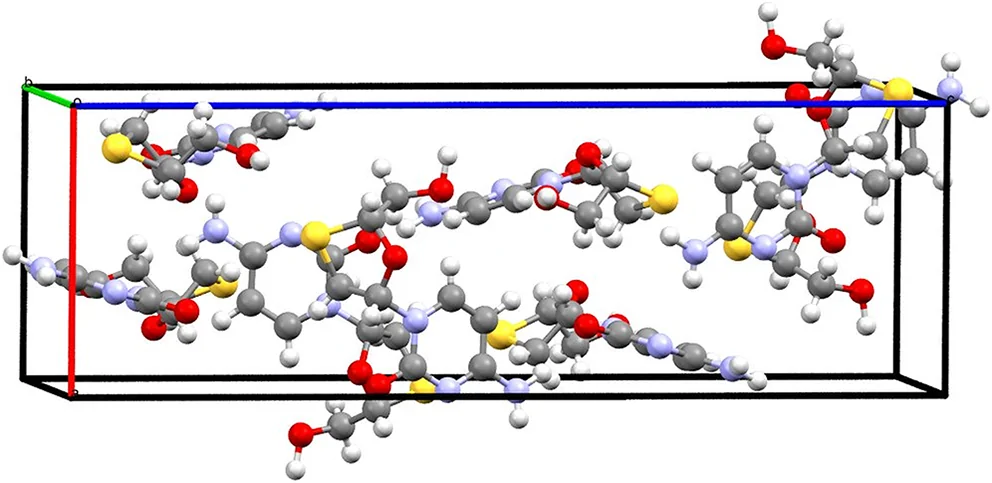
Representation of a unit cell containing eight molecules of lamivudine Form II.

NMR is a powerful method for the elucidation of crystal structures. NMR crystallography has received prestige over the past decade, especially due to the notable development of the solid-state NMR (ssNMR) technique and the appeal for featuring drugs directly in the solid-state forms incorporated into clinical formulations. The advances in studies of solid drugs via NMR, in connection with other techniques, may even allow the insertion of NMR techniques in the official physicochemical quality control compendia of drugs in the near future.^9–13^

The most studied nuclei in ssNMR are ^13^C, ^15^N, ^17^O, ^27^Al, ^31^P, ^51^V, ^93^Nb, and ^29^Si. Some properties should be considered when choosing which techniques are most appropriate for a given nucleus, such as nuclear spin, Larmor frequency, natural abundance, sensitivity, gyromagnetic ratio, chemical shift range, quadrupole moments and its reference for chemical shift. Several physical factors cause enlargement of the ssNMR signals, which most often hamper structural characterization. To deal with this challenge, the data provided by a ssNMR spectrum can be complemented and better understood with the aid of theoretical calculations of NMR parameters.^14–18^ This tool is used when the chemical shift and spin–spin coupling constants are calculated and compared with existing experimental NMR data. If the two sets of NMR data are in agreement, the compound/solid form in question has a high probability of having the molecular/intermolecular structure used in the calculations; thus, this reinforces the structural assignment.^18–21^

The calculation of NMR parameters contributes to the assignment and spectral interpretation, and is confirmation of experimental NMR parameters. Additional information such as anisotropy, orientation of tensors, spectral prediction, and evaluation of experimental viability can also be obtained. Different methods for obtaining the magnetic shielding tensor have been developed: the Gauge Including Atomic Orbital (GIAO)^22,23^ has been widely used in recent years to help elucidate both solid and liquid compounds.^17,24–28^ It is possible to use GIAO with different levels of theory, such as MP2 and b3lyp, with a different basis set. Other computational methods now available for this purpose can be found in the Quantum ESPRESSO (QE) software package.^29,30^ QE is an open-source software that uses the density functional theory (DFT), density functional perturbation theory (DTPT), and many-body perturbation theory (MBPT) based in plane wave pseudopotential, as well as projector augmented wave approaches, to simulate a wide range of material properties, such as energy, structure at the atomic level, vibrational properties, electronic response properties, and, in particular, a range of spectroscopic data, such as infrared and Raman.^31,32^ For the calculation of NMR properties, especially for ssNMR, QE has the Gauge Including Projected Augmented Wave (GIPAW)^33^ module, which is based in the plane wave pseudopotential formalism and avoids the use of cluster approximation.^34^ Information obtained by the GIPAW QE module allows calculation of the NMR shielding tensors and electric field gradient tensors, with a high precision, for various types of materials, including organic molecules, ceramics, and semiconductors.^14,35,36^

Here we have evaluated two theoretical approaches (GIPAW and GIAO) to accurately predict the ssNMR spectrum of three salts of lamivudine: lamivudinium chloride (**B**), lamivudinium salicylate monohydrate (**C**), and lamivudinium hydrogen phthalate (**D**), alongside lamivudine form II (**A**). These compounds have attracted considerable attention from the pharmaceutical industry owing to their improved physicochemical and pharmacological properties, which are highly relevant to drug discovery and formulation.^37^ Lamivudinium salicylate monohydrate (**C**) is a salt of lamivudine involving proton transfer that serves as a solid-state precursor for the preparation of the commercially marketed Form II. As a result of this study, computationally assisted ssNMR spectroscopy is shown to be a valuable tool for confirming the solid-state structures of pharmaceutical salts, with the potential to complement or supplement neutron and single-crystal X-ray diffraction data when these are unavailable.

## 2 Experimental Section

### 2.1 Preparation

#### 2.1.1 Lamivudinium Hydrogen Phthalate and Lamivudinium Salicylate Monohydrate

For the crystallization batches of each salt, an amount of lamivudine form II (10 mg, 0.04 mmol), whose authenticity and purity were previously checked by single-crystal and powder X-ray diffraction techniques, was dissolved in isopropyl alcohol (5 mL) with stirring for 5 min in a water bath (308 K). This solution was then allowed to cool slowly to room temperature (298 K). Next, an equimolar quantity of the counterion (7 mg of either phthalic acid or salicylic acid) was then added to the newly cooled solution. The resulting mixture was shaken at room temperature until the salt was completely dissolved. This solution was allowed to evaporate slowly upon standing for 5 days at 298 K. Crystals as prisms (lamivudinium hydrogen phthalate) or extremely thin needles (lamivudinium salicylate monohydrate) were formed on the bottom of the corresponding glass crystallizers.^6^

#### 2.1.2 Lamivudinium Chloride

Lamivudine form II was used as the starting material for preparation of 1 and 2. The authenticity and purity of lamivudine form II were first confirmed by single crystal and powder X-ray diffraction techniques before using it to prepare the crystalline modifications. For preparation of 1, a quantity of the drug (10 mg, 0.04 mmol) was dissolved in isopropanol (5 mL, 66 mmol) under stirring (5 min) on a water bath (308 K). The solution was then allowed to cool to room temperature. By adding a 0.28 mol L^–1^ solution of hydrochloric acid in water (0.25 mL, 0.07 mmol) to the preparation of the drug in isopropyl alcohol, crystals of 1 could be obtained after stirring (5 min, 298 K) and slow evaporation of the resulting solution upon standing (7 days, 298 K). On the other hand, by dissolution of lamivudine (10 mg, 0.04 mmol) in double-distilled water (5 mL, 0.28 mol) under shaking (10 min) on a water bath (313 K), crystals of 2 were obtained after addition of the same hydrochloric acid solution (0.25 mL, 0.07 mmol) to the aqueous solution of lamivudine, cooling to room temperature, stirring of the mixture (5 min, 298 K) and standing for 8 days at room temperature.^8^

### 2.2 Single-Crystal Data and Structural Confirmation by PXRD

The single-crystal data parameters of the four forms studied in this work are summarized in Table 1. All forms have *Z*′ = 1, that is, one crystallographically distinct lamivudine molecule (or ion pair) per asymmetric unit. The CIF files used as input for the GIPAW and GIAO calculations were retrieved from the Cambridge Structural Database (CSD)^38^ under the refcodes indicated.

**Table 1:** Single-Crystal Data Parameters for the Four Lamivudine Forms Studied in This Work, Extracted Directly from the CIF Files Used as input for the GIPAW and GIAO Calculations.^a^

	**form A** ^4,5^	**form B** ^8^	**form C** ^6^	**form D** ^6^
**parameter**	(Lamivudine Form II)	(Lamivudinium chloride)	(Lamivudinium salicylate monohydrate)	(Lamivudinium hydrogen phthalate)
empirical formula	C_8_H_11_N_3_O_3_S	C_8_H_12_N_3_O_3_S^+^ ·Cl^–^	C_8_H_12_N_3_O_3_S^+^ ·C_7_H_5_O_3_^–^ ·H_2_O	C_8_H_12_N_3_O_3_S^+^ ·C_8_H_5_O_4_^–^
formula weight (g mol^–1^)	229.26	265.72	385.40	395.39
crystal system	tetragonal	monoclinic	monoclinic	orthorhombic
space group	*P*4_3_2_1_2	*P*2_1_	*P*2_1_	*P*2_1_2_1_2_1_
*a* (Å)	8.7490(12)	5.6520(3)	12.7246(12)	5.39900(10)
*b* (Å)	8.7490(12)	11.9240(6)	5.2978(3)	15.7550(4)
*c* (Å)	26.523(5)	8.3220(6)	13.1688(9)	20.6980(5)
α (°)	90	90	90	90
β (°)	90	104.760(3)	106.129(8)	90
γ (°)	90	90	90	90
volume (Å^3^)	2030.2	542.3	852.8	1760.6
*Z*/*Z*′	8/1	2/1	2/1	4/1
temperature (K)	298	150	107	298
CSD refcode	RUKHAG	LASZAI01	GAXFUI	GAXFOC

a The CIFs are deposited in the GitHub repository accompanying this manuscript. All forms have *Z*′ = 1, that is, one crystallographically distinct lamivudine molecule (or ion pair, in forms B–D) per asymmetric unit. Superscripts on each Form heading indicate the original publication reporting the corresponding single-crystal structure.

Phase identity of all four crystalline forms was confirmed by powder X-ray diffraction (PXRD). The experimental PXRD patterns for lamivudinium hydrogen phthalate and lamivudinium salicylate monohydrate, as well as the corresponding theoretical diffractograms calculated from the single-crystal structures using Mercury,^39^ were reported previously by Capeletti da Silva et al.^6^ The experimental and theoretical PXRD patterns for lamivudinium chloride were similarly reported by Ellena et al.^8^ The PXRD pattern for lamivudine Form II is fully consistent with the published crystal structure.^4,5^ The experimental and calculated PXRD patterns of the four lamivudine forms used in this work, including overlays of the experimental diffractograms with the patterns calculated from the published single-crystal structures, are provided in Figure S1 of the Supporting Information. The previously published experimental PXRD data (refs 4–6,8 ) were used in this work exclusively to confirm phase purity prior to ssNMR and computational analyses.

### 2.3 Solid-State NMR Experiments

The ^13^C ssNMR spectra of lamivudine form II, lamivudinium chloride, lamivudinium salicylate-monohydrate and lamivudinium hydrogen phthalate were acquired on a Bruker Avance III 11.75 T operating at 500.13 MHz for ^1^H and 125.77 MHz for ^13^C, equipped with a 4 mm CP/MAS probe. The ^13^C reference chemical shift was set by the adamantane sample, with the value of 38.48 ppm to the more unshielded carbon. All samples were packed in a 4 mm zirconia rotor and the ^13^C ssNMR experiments were acquired using Cross-Polarization Total Suppression of Spinning Side Bands - CPTOSS - pulse sequence, a high-power ^1^H decoupling scheme, at 298 K. The main acquisition parameters were as follows: recycle delay was 4.0 s, contact time was 2.0 ms, acquisition time was 41.0 ms, spinning rate of 5.0 kHz, 12,000 scans, spectral window of 397.5 ppm, and 4096 data points.

A spinning rate of 5 kHz was chosen as the validated standard operating regime for the CPTOSS pulse sequence on a 4 mm CP/MAS probe. CPTOSS, originally introduced by Dixon,^40^ uses a train of four 180° pulses timed at specific fractions of the rotor period to refocus all spinning-sideband orders simultaneously and produce a sideband-free isotropic spectrum directly; the timing scheme is well-defined at moderate spinning rates (∼3–8 kHz) and becomes less robust at higher rates because of finite-pulse-width effects. CPMAS at faster spinning rates (e.g., 9.5 kHz, ref 27) is a valid alternative experimental design but produces residual sidebands that must be identified separately, which is why CPTOSS at 5 kHz was preferred here, where the goal is unambiguous assignment of isotropic ^13^C chemical shifts for direct comparison with GIPAW and GIAO calculated isotropic shielding tensors.

### 2.4 Calculations of NMR Parameters

Theoretical chemical shifts were obtained from the isotropic shielding tensor using Quantum ESPRESSO (QE) code for DFT-GIPAW^29^ and Gaussian09 for GIAO. In the GIPAW calculations, norm-conserving pseudopotentials were employed to describe the core electron wave functions; the *pbe-tm-new-gipaw-dc* pseudopotential was used for all atoms. The self-consistent field (SCF) convergence threshold, *conv*_*thr*, was set to 10^–7^ Ry and the plane-wave kinetic energy cutoff, *ecutwfc*, was set to 100 Ry (1360.6 eV). The k-point grid was set to 2 × 2 × 2. All GIPAW calculations were performed with Quantum ESPRESSO version 6.3. For the GIAO calculations, the geometry of each structure was first optimized at the B3LYP/cc-pVTZ level of theory.

In both approaches, the same Crystallographic Information File (CIF) obtained from the Cambridge Structural Database (CSD)^38^ was used to generate the input files.

## 3 Results and Discussion

### 3.1 Identity of the Four forms

All three binary phases **B**–**D** studied in this work are formally salts, in line with the original publications.^6,8^ For the systems studied here: form **B** (lamivudinium chloride, Δp*K*_a_ ≈ 11) and form **C** (lamivudinium salicylate monohydrate, Δp*K*_a_ ≈ 1.3) are canonical salts, with the bridging proton located on the cytosine N3 in the published single-crystal X-ray analyses;^6,8^ form **D** (lamivudinium hydrogen phthalate, Δp*K*_a_ ≈ 1.4 for the first deprotonation) is also a salt of the cytosine, but, in addition, the hydrogen phthalate anion contains an internal, near-symmetric, proton-shared O···H···O bond between the carboxyl and carboxylate groups, evidenced by the QTAIM bond critical points ID1 and ID2 discussed in section 3.4 (Table 3), placing form **D** between the canonical-salt and proton-shared-cocrystal extremes of the salt–cocrystal continuum.^41^

### 3.2 Methodological Scope: Why ^13^C Alone

The methodological choice of ^13^C as the sole nucleus for the GIPAW/GIAO benchmark is deliberate. ^13^C provides 8–16 chemically distinct sites per form (47 in total across forms **A**–**D**), spanning ∼140 ppm of chemical-shift range. This density of independent observations enables rigorous parametric and rank-based statistical inference (Shapiro–Wilk normality testing, followed by significance testing of Pearson’s *R* and Kendall’s τ at the 95% level). ^15^N, by contrast, would offer only three nitrogen environments per lamivudine molecule (the two ring nitrogens of the cytosine moiety and the exocyclic amino nitrogen) and none in the salicylate or phthalate coformers, giving 12 data points in total; with *n* = 3 per form, neither distributional testing nor correlation-based discrimination between GIPAW and GIAO is feasible. The proton positions in the bridging hydrogen bonds, which are central to the salt/cocrystal question, are independently established by single-crystal X-ray diffraction^6,8^ for forms **B** and **C** and, for the proton-shared O···H···O bridges of form **D**, by the QTAIM topology of the electron density (Table 3); ^1^H-detected and ^1^H–X HETCOR/PISEMA experiments would require ultrafast MAS hardware not used in this study and are outside the scope of this benchmark.

### 3.3 Solid-State NMR

The ^13^C CPTOSS NMR spectra of the lamivudine forms were acquired under magic angle spinning (MAS) conditions using cross-polarization (CP) combined with the Total Suppression of Spinning Sidebands (TOSS) technique. All four forms have *Z*′ = 1 (Table 1), so the multiplicity of ^13^C signals reflects the number of chemically distinct carbons in a single asymmetric unit (8 for forms **A** and **B**, 15 for form **C**, and 16 for form **D**), without additional peak splitting due to crystallographically inequivalent molecules.

Form **A** is the parent material from which the salts **B**, **C**, and **D** are derived (Figure 2). Solid-state NMR provides a direct means of confirming the formation of the salts: if a new solid phase has formed, the chemical shifts of **A** are expected to change, since both components occupy the same asymmetric unit and interact with each other. Conversely, if no shift changes are observed relative to pure **A**, the two components coexist as a simple physical mixture in separate asymmetric units.

**Figure 2:**
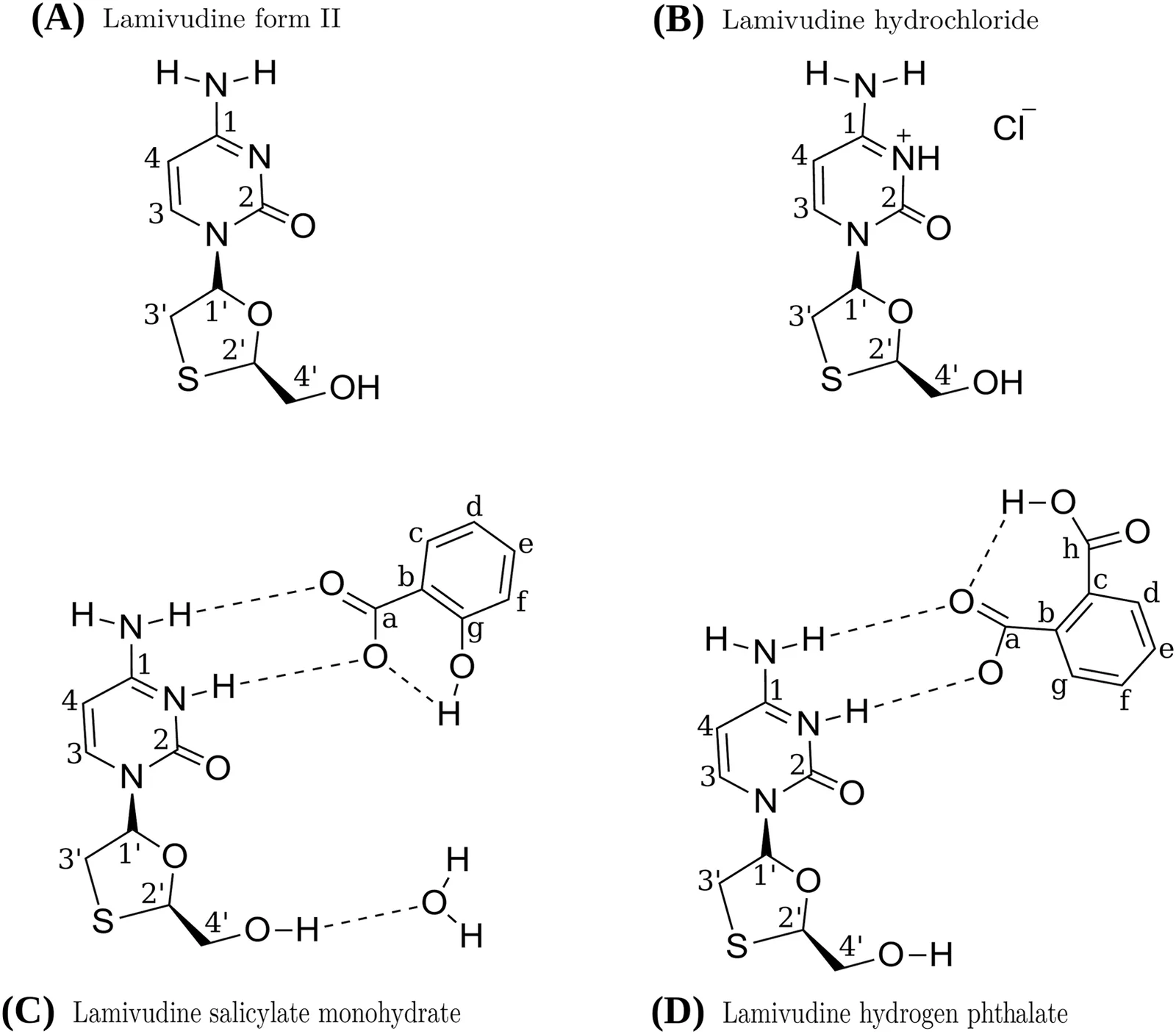
Lamivudine Form II (A) and its three salts (B–D) with carbon numbering labels used throughout the text.

When comparing the ^13^C CPTOSS NMR spectra of **A** with both **C** and **D** Figure 3, chemical shifts assigned to drug are very different from those reported for form II of lamivudine, which were also measured here and are in agreement with literature precedent.^42^ This is due to protonation of lamivudine and its typical conformation in the salt monohydrate differing from that in form II. The additional chemical shifts found only in the spectrum of **C** and **D** are attributed to the salicylate and the phthalate carbons, respectively (Figure 3).

**Figure 3:**
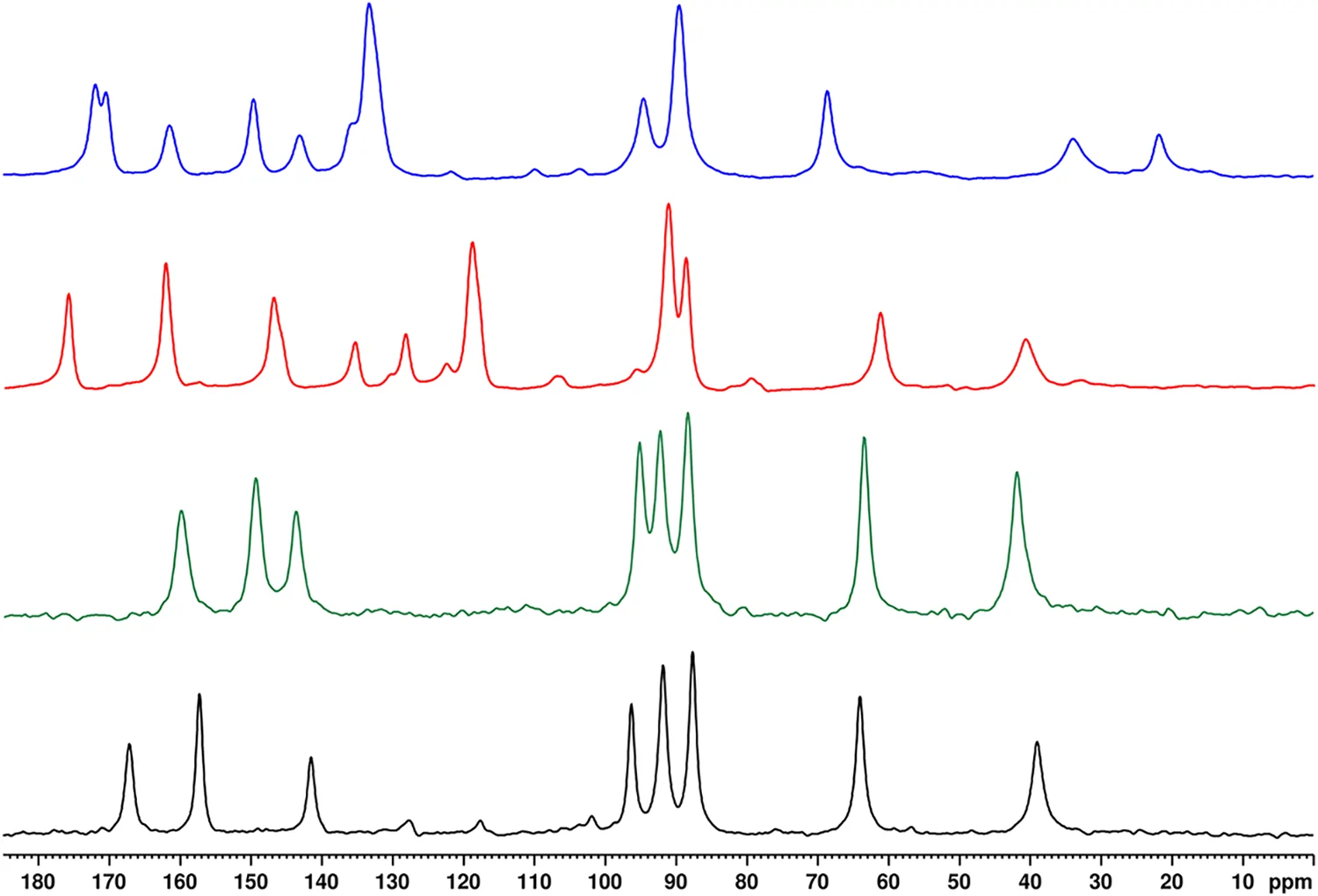
Spectra of lamivudine and the three salts: lamivudine Form II (black), lamivudinium chloride (green), lamivudinium salicylate monohydrate (red), lamivudinium hydrogen phthalate (blue).

The ^13^C chemical shift differences between **A** and forms **C** (ΔδC1:5.1 ppm; C2:10.0 ppm) and **D** (ΔδC1:5.7 ppm; C2:7.7 ppm), as reported in Table 2, arise from hydrogen bonds between the oxygen atoms of the acidic groups of salicylate (**C**) and hydrogen phthalate (**D**) and the N–H protons of the lamivudine cytosine ring.

**Table 2:** Experimental and Theoretical (GIPAW and GIAO) Chemical Shifts, Mean and Median of Module of Chemical Shifts Variation and Correlation Coefficient of Pearson and Kendall for all Lamivudine Forms^a^

**Lamivudine form II**						**Lamivudine hydrochloride**					
		**GIPAW**		**GIAO**				**GIPAW**		**GIAO**	
^13^ **C**	**δ**_exp_	**δ**_calc_	|δ_exp_ – **δ**_calc_|	**δ**_calc_	|δ_exp_ – **δ**_calc_|	^13^ **C**	**δ**_exp_	**δ**_calc_	|δ_exp_ – **δ**_calc_|	**δ**_calc_	|δ_exp_ – **δ**_calc_|
1	167.2	168.2	1.0	164.6	2.6	1	159.8	160.9	1.1	158.4	1.4
2	157.3	159.7	2.4	153.1	4.2	2	149.3	149.9	0.5	146.1	3.2
3	141.5	142.6	1.1	143.3	1.8	3	143.6	141.4	2.2	140.8	2.8
4	96.3	94.9	1.5	86.8	9.5	4	95.2	93.6	1.6	72.6	22.7
1′	91.8	86.7	5.2	90.4	1.4	1′	88.3	90.6	2.3	84.8	3.5
2′	87.7	84.4	3.3	91.2	3.5	2′	92.2	93.6	1.4	83.8	8.4
3′	39.1	26.6	12.5	36.8	2.3	3′	41.9	29.3	12.6	18.7	23.2
4′	64.1	47.0	17.2	65.5	1.4	4′	62.5	47.6	14.9	42.0	20.5
		***R* = 0.997**	**Δ̃** = 2.9	***R* = 0.996**	**Δ̃** = 2.4			***R*** = 0.995	**Δ̃** = 1.9	***R* = 0.993**	**Δ̃** = 6.0
		**τ** = 1.000	**Δ̅** = 5.5	**τ** = 0.786	**Δ̅** = 3.3			**τ** = 1.000	**Δ̅** = 4.6	**τ** = 0.786	**Δ̅** = 10.7

**Lamivudine salicylate monohydrate**						**Lamivudine hydrogen phtalate**					
		**GIPAW**		**GIAO**				**GIPAW**		**GIAO**	
^13^ **C**	**δ**_exp_	**δ**_calc_	|δ_exp_ – **δ**_calc_|	**δ**_calc_	|δ_exp_ – **δ**_calc_|	^13^ **C**	**δ**_exp_	**δ**_calc_	|δ_exp_ – **δ**_calc_|	**δ**_calc_	|δ_exp_ – **δ**_calc_|
1	162.1	158.3	3.8	152.6	9.5	1	161.5	159.6	1.9	154.6	6.9
2	147.0	146.6	0.4	140.2	6.8	2	149.6	150.6	1.0	142.9	6.7
3	145.9	145.8	0.1	141.1	4.8	3	143.1	144.1	1.0	137.8	5.3
4	91.0	88.2	2.8	76.1	14.9	4	94.5	92.1	2.4	78.1	16.4
1′	91.4	88.7	2.7	79.6	11.8	1′	89.7	89.6	0.1	80.7	9.0
2′	88.7	96.3	7.6	87.8	0.9	2′	89.4	88.6	0.9	80.8	8.6
3′	40.8	33.8	7.0	26.2	14.6	3′	34.2	22.4	11.8	14.9	19.3
4′	61.3	46.9	14.4	38.3	23.0	4′	68.7	53.3	15.5	44.6	24.1
a	175.9	179.0	3.1	173.5	2.4	a	171.9	175.9	3.9	170.6	1.3
b	122.6	125.5	2.9	115.7	6.9	b	136.0	136.7	0.7	126.0	10.0
c	128.3	126.6	1.7	122.2	6.1	c	136.0	137.7	1.7	132.5	3.5
d	118.0	115.8	2.2	104.0	14.0	d	133.5	132.3	1.2	122.9	10.6
e	135.5	132.3	3.2	119.3	16.2	e	121.8	126.5	4.7	116.5	5.3
f	118.9	116.9	2.0	103.4	15.5	f	121.8	127.6	5.8	113.9	7.9
g	162.3	164.9	2.6	158.6	3.7	g	132.4	130.3	2.1	122.1	10.3
		***R* = 0.995**	**Δ̃** = 2.8	***R* = 0.993**	**Δ̃** = 9.5	h	170.4	171.1	0.7	160.9	9.5
		**τ** = 0.962	**Δ̅** = 3.8	**τ** = 0.905	**Δ̅** = 10.1			***R* = 0.996**	**Δ̃** = 1.8	***R*** = 0.996	**Δ̃** = 8.8
								**τ** = 0.992	**Δ̅** = 3.5	**τ** = 0.941	**Δ̅** = 9.7

a Δ̅: Mean. **R**: Pearson’s correlation coefficient. δ_exp_: Experimental Chemical Shift. Δ̃: Median. τ: Kendall’s correlation coefficient. δ_calc_: Theoretical Chemical Shift.

For form **B**, the shielding of ^13^C nuclei by the chloride counterion is most pronounced at carbons C1 and C2. The differences in chemical shift for these carbons in **B** relative to **A** are 7.4 and 8.0 ppm, respectively. Thus, it can be said that the chlorine atom, at the time of cocrystallization, is arranged close to these carbons.

Two theoretical methods were employed for chemical shift assignment, GIPAW and GIAO (Table 2). The GIPAW method is particularly advantageous for solid-state applications, as it accounts for the periodic crystallographic environment of the unit cell, whereas GIAO calculations use only the isolated molecular coordinates.

### 3.4 QTAIM and NBO

The formation of a salt requires the establishment of hydrogen bonds between the participating components at the moment of crystallization, resulting in a phase in which both species occupy the same crystallographic asymmetric unit. Having confirmed the formation of forms **B**–**D** by ssNMR, a systematic investigation of all possible inter- and intramolecular hydrogen-bonding interactions was carried out.

Different studies have pointed out that the formation of hydrogen bonds is associated with the appearance of a critical point (CP) of bond between the hydrogen atom and the donor atom, which are linked by the path of the concomitant bond.^43–46^ This critical point has typical interaction properties of closed-shell type, that is, it has the valence orbitals completely filled, leading to high bond stability. In another sense, a closed-shell configuration corresponds to a state in which all molecular orbitals are doubly occupied or empty (a singlet state).

To explain the character of the strength of hydrogen bonds, topological analysis was used following the Quantum theory of atoms in molecules.^47,48^ This theory is supported by calculations of electronic density (ρ­(*r*)), Energy density (*H*(*r*)) and Laplacian of electronic density (∇^2^ ρ­(*r*)) at the critical point. Thus, it was possible to identify possible interactions, inter- and intramolecular, and still classify the strength of the interactions as strong, medium or weak.

As demonstrated by Koch and Popelier,^45^ the electron density at the bond critical point increases with increasing hydrogen bond strength and describes the degree of charge concentration along the bond. The electron density at the bond critical point is inversely related to the donor–acceptor distance, so that shorter contacts correspond to stronger interactions. The distances of hydrogen bonds and the values of ρ­(*r*) are illustrated in Table 3.

**Table 3:** Values of Electronic Density (ρ (*r*)), Laplacian of Electronic Density (∇^**2**^ρ­(***r***)) and Energy Density (*H*(*r*)) for Classification of Interaction Force of all Hydrogen Bonds of Lamivudine Forms^a^

		**QTAIM**				**NBO**	
	***r*** ′_**i**–*j*_/Å	ρ(*r*)/u.a	∇^**2**^ρ(*r*)/u.a	***H*(*r*)**/u.a	**strength**	**Doação**	***E*** ^**(2)**^/kcal mol^–^
**IA1**	2.33	0.010035	0.040411	0.001898	weak	**η**_O_ → σ*(C–H)	0.85
**IB1**	2.59	0.011604	0.040021	0.002032	weak	**η**_Cl_ → σ*(N–H)	1.28
**IB2**	2.23	0.023439	0.081330	0.001847	weak	**η**_Cl_ → σ*(N–H)	6.22
**IC1**	1.83	0.034709	0.150776	0.001438	weak	**η**_O_ → σ*(O–H)	5.23
**IC2**	1.95	0.025465	0.105594	0.002852	weak	**η**_O_ → σ*(N–H)	5.36
**IC3**	1.77	0.038495	0.144216	–0.000216	medium	**η**_O_ → σ*(N–H)	10.93
**IC4**	2.27	0.013645	0.051726	0.002222	weak	**η**_O_ → σ*(C–H)	0.91
**IC5**	1.90	0.026026	0.124298	0.003525	weak	**η**_O_ → σ*(O–H)	3.62
**IC6**	3.41	0.005643	0.014421	0.000278	weak	**η**_S_ → σ*(O–H)	0.16
**ID1**	1.17	0.194784	–0.573852	–0.233458	strong	**η**_O_ → η*(*H*)	330.61
**ID2**	1.23	0.155809	–0.132379	–0.134525	strong	**η**_O_ → η*(*H*)	223.20
**ID3**	1.92	0.026080	0.111004	0.003040	weak	**η**_O_ → σ*(N–H)	9.81
**ID4**	1.85	0.031205	0.130819	0.002465	weak	**η**_O_ → σ*(N–H)	10.06
**ID5**	2.80	0.010619	0.036416	0.001441	weak	**η**_S_ → σ*(C–H)	

a Distance of bonds are represented in Å ngström (*r*_*i*–*j*_^′^). The stabilization energy from NBO, for donations of type η → σ* and η → η*, are expressed in kcal mol^–^.

The interactions that have been observed are illustrated in the Figure 4. The classification proposed by Rozas et al:^49^ the hydrogen bond strength is defined as weak if ∇^2^ρ­(*r*) > 0 and *H*(*r*) > 0, medium strength if ∇^2^ρ­(*r*) > 0 and *H*(*r*) < 0 or strong bond if ∇^2^ρ­(*r*) < 0 and *H*(*r*) < 0.

**Figure 4:**
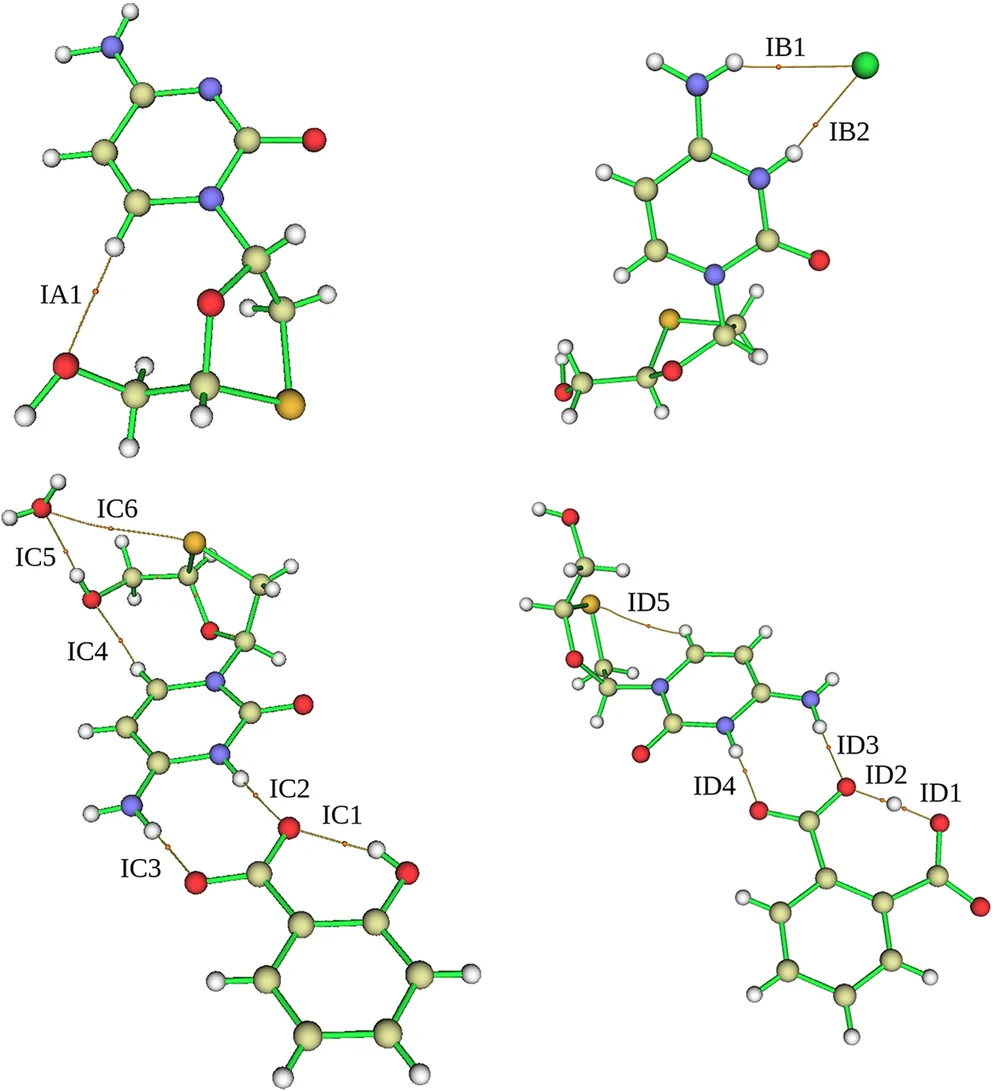
Molecular structures of the four lamivudine forms, with labeled inter- and intramolecular interactions identified by QTAIM analysis.

In the analysis of Natural Bond Orbital (NBO), the electron wave function is interpreted in terms of a set of delocalized Lewis orbitals.^50^ The delocalization energy, or stabilization energy *E* ^(2)^, was estimated by the second order Perturbation theory. Therefore, it was possible to measure the stabilization energies of electronic donations that occur between electron lone pairs (η) of oxygen, or sulfur, to orbitals of type σ*­(C–H), σ*­(N–H) and σ*­(O–H) for the case of intermolecular donation between S···O–H in **C** (**IC6** Figure 4).

Table 3 presents the values of ρ­(*r*), ∇^2^ρ­(*r*), and *H*(*r*) for all hydrogen bonds identified by the QTAIM method, together with the distance between the donor atom (i) and the acceptor (j) (*r*_*i*–*j*_^′^), and the stabilization energies calculated by second-order perturbation theory *E* ^(2)^ within the NBO framework.

Following the classification scheme of Rozas et al.,^49^ interactions **ID1** and **ID2** are classified as *strong* (∇^2^ρ­(*r*) < 0 and *H*(*r*) < 0); interaction **IC3** as *medium* (∇^2^ρ­(*r*) > 0 and *H*(*r*) < 0); and interactions **IA1**, **IB1**, **IB2**, **IC1**, **IC2**, **IC4**, **IC5**, **IC6**, **ID3**, **ID4**, and **ID5** as *weak* (∇^2^ρ­(*r*) > 0 and *H*(*r*) > 0). It was still possible to correlate these results with *r*_*i*–*j*_^′^, since the strong interaction, **ID1** and **ID2**, have the shortest connection distance being 1.17 and 1.23 Å respectively. The **IC3** interaction defined as moderate has *r*_*i*–*j*_^′^ equal to 1.77 Å being greater than the strong interactions and less than the weak hydrogen bonds, so the influence of interatomic distance is evident for the binding force.

Correlating ρ­(*r*) and the distances of hydrogen interactions with the bonding force, present in Table 3, it is also possible to infer a quantitative character of bonding force, since the interaction that has greater electronic density has greater bonding strength. The interactions **ID1** and **ID2** have in average a magnitude of electronic density at the critical point ten times greater than the other interactions, since the hydrogen is stabilized by resonance by the two oxygen atoms of the carboxylic acid group of Hydrogen phthalate. The donor–acceptor distances for **ID1** and **ID2** indicate that the bridging proton is nearly equidistant between the two oxygen atoms, consistent with a proton-shared (Zundel-type) strong hydrogen bond. The precise proton position, and whether a formal O–H covalent bond is present, are temperature-dependent; all calculations were performed at 298 K.

With the NBO analysis it was possible to identify all hydrogen interactions pointed out by QTAIM with the exception of the **ID5** bond, which consists of the electronic density donation of lone electron pairs of sulfur atom (η_*S*_) to the antibonding orbital σ*­(*C*–*H*), probably this donation was below the limit of 0.05 kcal mol^–1^ stipulated by the NBO calculation. This interaction has a relatively long *r*_*i*–*j*_^′^ of approximately 2.80 Å.

Hydrogen bonds involving sulfur atoms are longer than those involving nitrogen or oxygen, due to the size of the sulfur and its high polarizability, it is seen in Table 3 that interactions involving donations of sulfur atoms have *r*_*i*–*j*_^′^ equal to 3.41 and 2.80 Å for **IC6** and **ID5** respectively. In most cases, hydrogen bonds with sulfur atoms are long and classified as weak.^51–55^

In general, interactions with shorter donor–acceptor distances exhibit larger stabilization energies. The strongest interactions, **ID1** and **ID2**, have *E* ^(2)^ values of 330.61 and 223.20 kcal mol^–1^ and *r*_*i*–*j*_^′^ distances of 1.17 and 1.23 Å, respectively. These two interactions can be understood, according to the NBO, as the donation of oxygen lone pairs (η_*O*_) to the hydrogen antibonding valence orbital (η*­(*H*)), so there is no covalent bond formation between the hydrogen atom and one of the oxygen atoms.

Figure 5 compares the p*K*_a_ values for the first and second dissociation steps of the three isomers of phthalic acid.^56,57^ The anomalously low p*K*_a2_ of *ortho*-phthalic acid (5.41) relative to the *meta* (4.46) and *para* (4.82) isomers is a consequence of intramolecular hydrogen-bond stabilization of the monoanion after the first deprotonation. This is precisely the structural context of lamivudinium hydrogen phthalate and is consistent with the exceptionally large stabilization energies computed for the **ID1** and **ID2** interactions.

**Figure 5:**
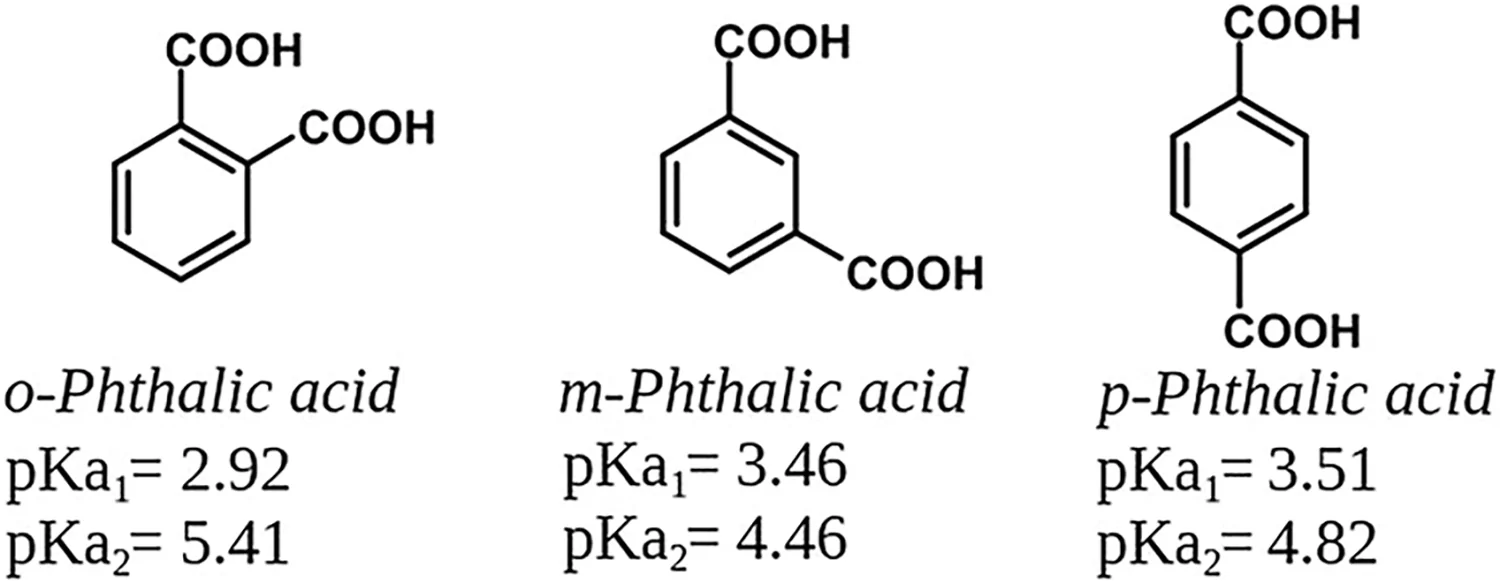
Phthalic acid: *ortho*, *meta*, and *para* with their first and second acid dissociation constants (p*K*_a_).

### 3.5 GIPAW and GIAO Comparison

The assignments for theoretical values of chemical shift for GIPAW and GIAO methods are referenced in Table 2. The GIPAW method is particularly well-suited for solid-state applications because it explicitly incorporates the periodic crystallographic conditions defined by the unit cell parameters, whereas the GIAO approach treats the molecule as an isolated species, using only atomic coordinates without periodic boundary conditions. Theoretical chemical shifts were obtained by subtracting the calculated isotropic shielding tensor (σ_iso_^calc^) from the experimentally determined reference shielding (σ_ref_^exp^), as given by eq 1. For GIPAW, the carbonyl carbon of glycine (σ_ref_^exp^ = 173.0 ppm) was used as the chemical shift reference, while for GIAO, tetramethylsilane (TMS, σ_ref_^exp^ = 174.3 ppm) served as the reference standard.

1 $$\delta_{\mathrm{calc}}=\sigma_{\mathrm{ref}}^{\exp}-\sigma_{\mathrm{iso}}^{\mathrm{calc}}$$

The following metrics were used to evaluate and compare the two approaches: the absolute error |δ_exp_ – δ_calc_|for each carbon, the mean (Δ̅) and median (Δ̃) of the absolute error distribution, Pearson’s correlation coefficient (*R*), and Kendall’s rank correlation coefficient (τ).

The median was chosen as the primary comparative metric because the absolute error distributions exhibited, in some cases, high variance and potential outliers (Figure 6). The box plots show that GIPAW forms **C** and **D**, and GIAO forms **A** and **D**, contain outlier absolute errors above the upper fence, and that form **B** shows particularly large dispersion for GIAO. Given this nonuniformity, the median is a more robust representative of the central error tendency than the mean. As expected, when the error distribution is approximately symmetric and outlier-free, as for GIAO **C**, the mean (10.1 ppm) and median (9.5 ppm) converge (Table 2).

**Figure 6:**
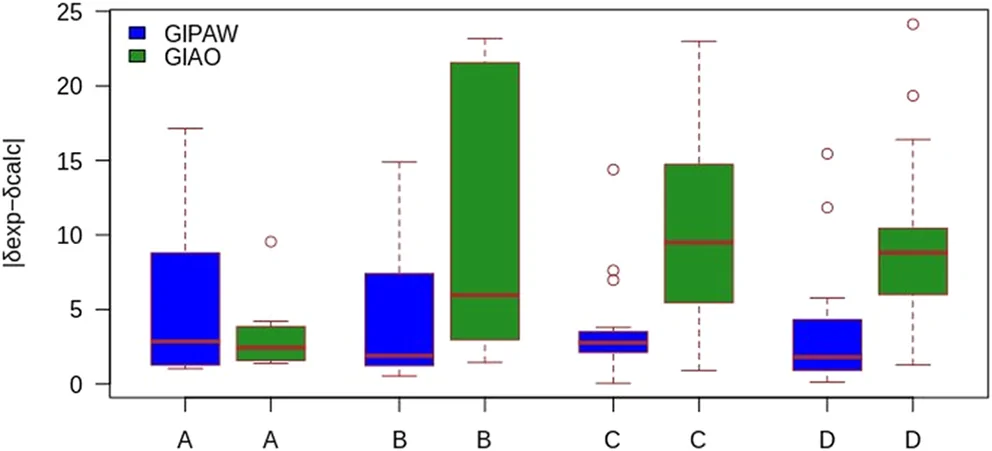
Box plots of the ^13^C chemical shift absolute errors for GIPAW (blue) and GIAO (green) methods across all four lamivudine crystalline forms (A–D). Circles represent statistical outliers.

The difference between the two methods, as well as their relation to the median is best seen in Figure 7 represented by the horizontal dashed line. For forms **B**, **C**, and **D**, the GIPAW method yielded smaller absolute errors, which is attributable to its periodic treatment of the crystallographic environment.^34,58,59^ The GIAO approach performed comparatively poorly for solids with significant intermolecular interactions, as it neglects the influence of neighboring molecules on the shielding tensor. For form **A**, although GIPAW has a smaller error for most carbons, the more pronounced deviations for C3′ and C4’ carbons in the GIPAW method, caused the overall median error for Form **A** to be greater with GIPAW than with GIAO.

**Figure 7:**
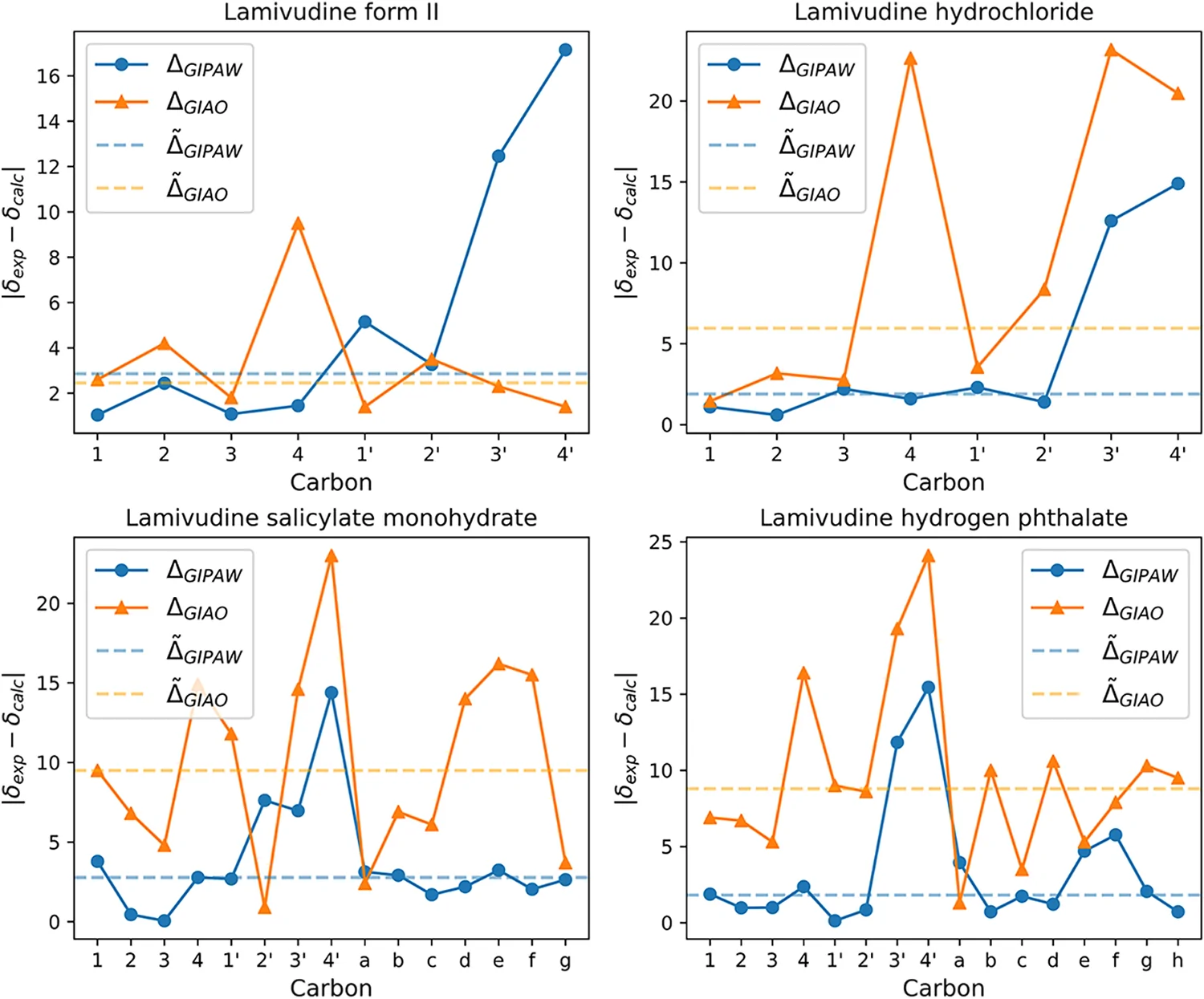
Per-carbon absolute errors |δ_exp_ – δ_calc_|for the GIPAW (Δ_GIPAW_, blue) and GIAO (Δ_GIAO_, orange) methods for all four lamivudine crystal forms. Dashed horizontal lines indicate the respective median errors (Δ̃).

A prerequisite for the application of parametric correlation tests is that both data sets follow a normal distribution. This was verified using the Shapiro–Wilk normality test^60,61^ at the 95% confidence level. The resulting p-values (Table 4, row “p-value Norm”) are all greater than 0.05, indicating that the null hypothesis of normality cannot be rejected. Both the experimental and theoretical ^13^C chemical shift data sets therefore satisfy the conditions for the application of Pearson’s and Kendall’s correlation tests.

**Table 4:** *P*-values from Normalization and Significance Tests to Chemical Shifts for Both Forms of Lamivudine^a^

	**A**_exp_	**B**_exp_	**C**_exp_	**D**_exp_	**A**_GIPAW_	**B**_GIPAW_	**C**_GIPAW_	**D**_GIPAW_	**A**_GIAO_	**B**_GIAO_	**C**_GIAO_	**D**_GIAO_
*p*-value Norm	0.5989	0.4625	0.6838	0.2845	0.548	0.4593	0.576	0.1349	0.4832	0.4928	0.7532	0.2456
*p*-value *R*					3.7 *×* 10^–8^	3.7 *×* 10^–7^	4.1 *×* 10^–14^	6.3 *×* 10^–16^	1.7 *×* 10^–7^	6.9 *×* 10^–7^	2.5 *×* 10^–13^	5.2 *×* 10^–16^
***p*-value** τ					0.00084	0.0012	7.1 *×* 10^–7^	1.2 *×* 10^–7^	0.0094	0.0094	3.3 *×* 10^–6^	5.9 *×* 10^–7^

a *p-*value Norm: *p*-values of Shapiro-Wilk normality test. *p-*value *R*: *p*-values of significance test for Pearson’s correlation coefficients. *p-*value τ: *p*-values of significance test for Kendall’s correlation coefficients.

Analyzing only Pearson’s correlation coefficients,^62^ Table 2, of ^13^C chemical shifts simulated by GIPAW and GIAO, both methods appear to be equivalent. The significance of Pearson’s coefficients was calculated to a significance level of 95% using the cor.test function of the *R* language, thus being all p-values, Table 4 (*p*-value *R*), are less than 0.05 so Pearson’s coefficients are statistically significant.

Although the Pearson correlation coefficients are statistically significant, it was insufficient to discriminate which of the two methods, GIPAW or GIAO, best describes the experimental condition. To discriminate between the two methods, Kendall’s rank correlation coefficient (τ)^63^ was therefore employed as a complementary metric. This coefficient is a measure of the monotonic correlation strength between two sets of variable, experimental and theoretical in this case. The significance test for this coefficient was calculated using the Kendall package of the R language. The null hypothesis for this test is *H*_0_: x and y are independent. Adopting a significance level of 95% it is possible to notice that the theoretical variables, both GIPAW and GIAO, are dependent when related to the experimental variables, since the p-value described in Table 4 (*p*-value τ), is less than 0.05, so we reject the null hypothesis.

The Kendall τ coefficient measures the degree of concordance between two ordered sequences. A value of τ = 1 indicates perfect agreement, meaning that ranking the experimental shifts in ascending order produces the identical ordering of the theoretical values. This perfect concordance was achieved for form **A** with GIPAW and for form **B** with GIAO, reflecting an ideal correspondence between predicted and experimental shift sequences for those cases.

Several benchmark studies comparing GIPAW and GIAO ^13^C chemical shifts have previously been reported, with system-dependent rankings between the two methods. Szeleszczuk et al. showed that for eight variations of cinnamic acid, GIPAW was more accurate.^58^ On the other hand, Marín-Luna et al. demonstrated that for two crystalline structures of Benzimidazole, four Indazole, and three Benzotriazole, calculations using the GIAO theory were more accurate.^64^ The relative performance of the two methods is therefore not universal but depends on whether intermolecular contacts dominate the shielding (favoring GIPAW) or the local electronic structure dominates (where extended-basis GIAO can be competitive). The four lamivudine forms studied here span this spectrum: form **A** is dominated by intramolecular geometry, while forms **C** and **D** feature strong intermolecular hydrogen bonds. In this work, the GIPAW method proved to be more efficient for all studied crystalline forms of lamivudine, being proven by the lower values of median errors, and even more agreement between the theoretical and experimental data, confirmed by the Kendall’s coefficient.

## 4 Conclusions

The ^13^C ssNMR spectra of the Lamivudinium chloride, Lamivudinium salicylate monohydrate and Lamivudinium hydrogen phthalate were recorded for the first time and the spectrum of Lamivudine form II is in accordance with the literature.^42^ All four crystalline forms of lamivudine studied here are pharmaceutically relevant, as they possess potential antiviral activity for the treatment of HIV/HBV.

The ^13^C NMR chemical shifts of all four crystalline forms, lamivudine Form II, lamivudinium chloride, lamivudinium salicylate monohydrate, and lamivudinium hydrogen phthalate, were computed using both the GIPAW and GIAO approaches. Experimental chemical shift reference values of glycine carbonyl (173.0 ppm, GIPAW) and tetramethylsilane (174.3 ppm, GIAO) were used to convert the calculated isotropic shielding tensors into theoretical ^13^C chemical shifts.

A rigorous statistical evaluation, including Shapiro–Wilk normality testing and significance tests for both Pearson’s *R* and Kendall’s τ, confirmed the validity of the comparison. On the basis of median absolute errors and Kendall’s τ, the GIPAW method proved superior to GIAO for all four lamivudine crystal forms investigated, a result attributable to its periodic, plane-wave formalism that faithfully captures the solid-state environment. These findings support the wider adoption of GIPAW-based NMR calculations in the structural characterization of pharmaceutical salts.

## Supplementary Material



## Data Availability

The Quantum ESPRESSO (GIPAW) and Gaussian 09 (GIAO) NMR files, together with the NBO analysis and crystallographic information files (CIFs) used to generate the computational input data, are openly available in a public GitHub repository at https://github.com/luizkeng/lamiv_data. All software used in this work is publicly available: Quantum ESPRESSO (open-source, https://www.quantum-espresso.org), Gaussian 09 (commercial, https://gaussian.com), and R (open-source, https://www.r-project.org).

## Notes

Experimental and calculated powder X-ray diffraction patterns of the four lamivudine forms studied in this work, lamivudine Form II, lamivudinium chloride, lamivudinium salicylate monohydrate, and lamivudinium hydrogen phthalate, with the corresponding measurement and simulation parameters (Figure S1) (PDF)
