# Supplementary material for: Combining X‑ray Diffraction, Solid-State NMR, and Computational Chemistry for Structural Insights and Performance Evaluation of GIPAW/GIAO 13C Chemical-Shift Calculations for Lamivudine and Three Pharmaceutical Salts
Source: ACS Omega. 2026 Sep 2;11(36):53927–37. doi: 10.1021/acsomega.6c03134 (PMC13584537; doi:10.1021/acsomega.6c03134)
Supplement: Supplementary file 1 [file ao6c03134_si_001.pdf]

Supporting Information

Combining X-ray Diffraction, Solid-State NMR, and Computational Chemistry for Structural Insights and Performance Evaluation of GIPAW/GIAO ^{13}C Chemical-Shift Calculations for Lamivudine and Three Pharmaceutical Salts

Jefferson R. Dias-Silva,[a] Vinícius S. Ferreira,[a,b] Felipe T. Martins,[c] Valdemar Lacerda Júnior,[d] Pedro L. de Souza,[a] Thiago M. F. Marinho,[a] and Luiz H. K. Queiroz Júnior[a,*]

[a] *Instituto de Química, Universidade Federal de Goiás, CP 131, 74001-970, Goiânia-GO, Brazil*

[b] *Instituto Federal de Goiás, R. 75 n. 46, 74055-110, Goiânia-GO, Brazil*

[c] *Instituto de Química, 37130001, Alfenas-MG, Brazil*

[d] *Departamento de Química, Centro de Ciências Exatas, Universidade Federal do Espírito Santo, 29075-910, Vitória-ES, Brazil*

[*] *Corresponding author: keng@ufg.br*

Contents

- Figure S1 — Experimental and calculated PXRD patterns of the four lamivudine forms studied in this work.

Figure S1. Experimental and calculated PXRD patterns of the four lamivudine forms

Room temperature (298 K) experimental X-ray powder diffractograms were measured on a Shimadzu XRD-6000 diffractometer (Cu Kα beam generated at 40 kV and 30 mA, $\lambda = 1.5418$ Å). The continuous θ–2θ scan mode was used at a speed of 1.000° min^{-1}, with a step of 0.020° in a 2θ interval between 10° and 40°.

The calculated powder X-ray diffraction (PXRD) patterns of the four lamivudine forms shown in Figure S1 were simulated directly from the Crystallographic Information Files (CIFs) used as input for the GIPAW and GIAO chemical-shift calculations described in the main manuscript. The simulations were performed with the `XRDCalculator` module of PYMATGEN[1] using the standard structure-factor formalism, with the following parameters: Cu Kα_1 wavelength ($\lambda = 1.5418$ Å), 2θ range 10–40° in steps of 0.02°, and a pseudo-Voigt peak profile with FWHM 0.10°. The same patterns are obtainable in Mercury[2] from the same input CIFs. The overlay between the experimental diffractograms and the patterns calculated from the published single-crystal structures (CSD refcodes RUKHAG, LASZAI01, GAXFUI, and GAXFOC for lamivudine Form II, lamivudinium chloride, lamivudinium salicylate monohydrate, and lamivudinium hydrogen phthalate, respectively) confirms phase identity and

[1] S. P. Ong *et al.*, *Comput. Mater. Sci.* **2013**, *68*, 314–319; pymatgen.org.

[2] C. F. Macrae *et al.*, *J. Appl. Crystallogr.* **2020**, *53*, 226–235; www.ccdc.cam.ac.uk/solutions/csd-core/components/mercury.

bulk purity for all four samples used in the ssNMR experiments. The CIFs used to generate Figure S1 are openly available in the GitHub repository accompanying this work, at https://github.com/luizkeng/lamiv_data.

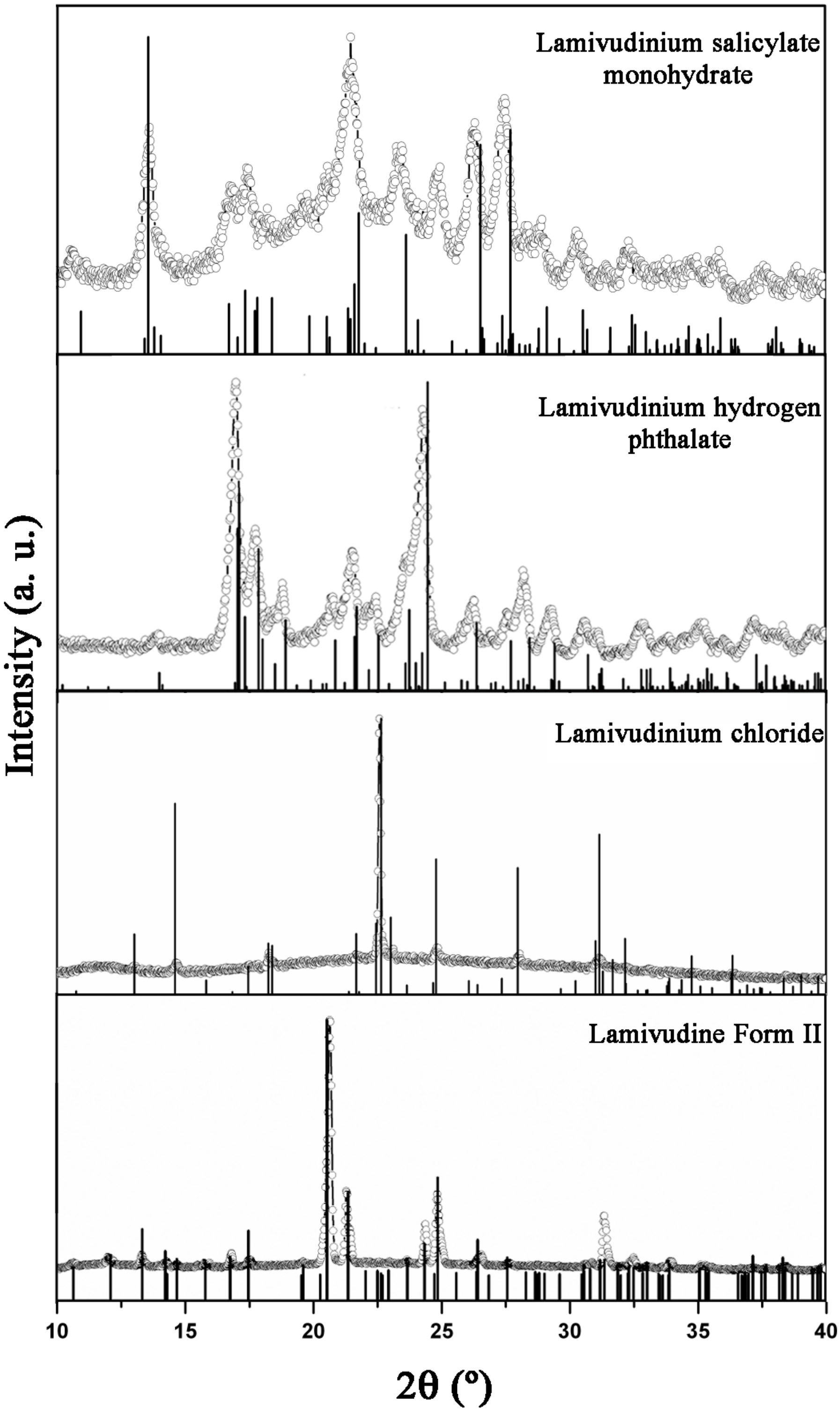

Figure S1: Experimental (open circles, ○) and calculated (vertical lines, simulated with the `XRDCalculator` module of PYMATGEN: Cu Kα_1, $\lambda = 1.5418$ Å, 2θ range 10–40°, step 0.02°, pseudo-Voigt FWHM 0.10°) powder X-ray diffraction patterns of the four lamivudine forms studied in this work: from top to bottom, lamivudinium salicylate monohydrate (CSD refcode GAXFUI; monoclinic $P2_1$), lamivudinium hydrogen phthalate (CSD refcode GAXFOC; orthorhombic $P2_12_12_1$), lamivudinium chloride (CSD refcode LASZAI01; monoclinic $P2_1$), and lamivudine Form II (CSD refcode RUKHAG; tetragonal $P4_32_12$). The same CIFs were used as input for the GIPAW (Quantum ESPRESSO) and GIAO (Gaussian 09) chemical-shift calculations reported in the main manuscript.
